# Atractylenolide II Inhibits Proliferation, Motility and Induces Apoptosis in Human Gastric Carcinoma Cell Lines HGC-27 and AGS

**DOI:** 10.3390/molecules22111886

**Published:** 2017-11-03

**Authors:** Shuang Tian, Hongdan Yu

**Affiliations:** Department of Cell Biology, Jinzhou Medical University, Jinzhou 121000, China; yuhongdan1116@126.com

**Keywords:** Atractylenolide II, gastric carcinoma, proliferation, apoptosis, motility

## Abstract

Atractylenolide II (AT-II) exhibits several biological and pharmacological functions, especially anti-cancer activity as the major sesquiterpene lactones isolated from *Atractylodes macrocephala* (also named *Baizhu* in Chinese). However, the effects and mechanisms of AT-II on human gastric cancer remain unclear. Cell Counting Kit-8 (CCK-8) assay, morphological changes, flow cytometry, wound healing assay and Western blot analysis were used to investigate the effects of AT-II on cell proliferation, apoptosis and motility of human gastric carcinoma cell lines HGC-27 and AGS. Our results indicated that AT-II could significantly inhibit cell proliferation, motility and induce apoptosis in a dose and time-dependent manner. Western blot analysis showed that the expression level of Bax was upregulated and the expression levels of B-cell lymphoma-2 (Bcl-2), phosphorylated-protein kinase B (p-Akt) and phosphorylated-ERK (p-ERK) were downregulated compared to control group. In conclusion, the findings suggested that AT-II exerted significant anti-tumor effects on gastric carcinoma cells by modulating Akt/ERK signaling pathway, which might shed light on therapy of gastric carcinoma.

## 1. Introduction

Gastric cancer is the fifth most common cancer and the second leading cause of cancer-related deaths in the world [1,2]. Although advanced surgeries and chemotherapy treatments are the most effective therapeutic methods for gastric cancer, there is still a high death rate and a low survival rate [3,4,5]. Therefore, it is urgent to find a novel agent that could improve the survival rate and alleviate the pain of gastric cancer patients.

Nowadays, more and more naturally bioactive compounds found in vegetables [6], pharmaceutical plants [7,8] and fruits are given special attention by researchers because of their high activity and low cytotoxicity. *Atractylodes macrocephala* belongs to the composite family has been an important traditional herbal medicine in Asia, which is widely used to treat dyspepsia, diarrhea, stomach diseases, diabetes and anti-abortion [9,10,11,12]. It is also popularly used as heath cultivating food. There are a large number of natural compounds that extracted from *Atractylodes macrocephala*, such as polysaccharides, sesquiterpenoids and volatile oils [13]. AT-II as the major sesquiterpenoids isolated from the dried Rhizome of *Atractylodes macrocephala* shows a wide range of biological and pharmacological activities, for example, against insomnia and anxiety, neuroprotective, platelet activation and anti-cancer effect [14,15,16,17]. Previous studies reported that AT-II could inhibit cell proliferation, arrest G1 phase cell cycle and induce apoptosis in B16 cell [18]. However, the effects and mechanisms of AT-II on human gastric cancer remain elusive.

The purpose of our study is to investigate the effects of AT-II on cell proliferation, motility and apoptosis of gastric carcinoma cells and its possible molecular mechanisms, which would provide valid data for the application of AT-II to treat gastric carcinoma in the future. 

## 2. Results

### 2.1. AT-II Inhibits Proliferation in HGC-27 and AGS Cells

**Figure 1:**
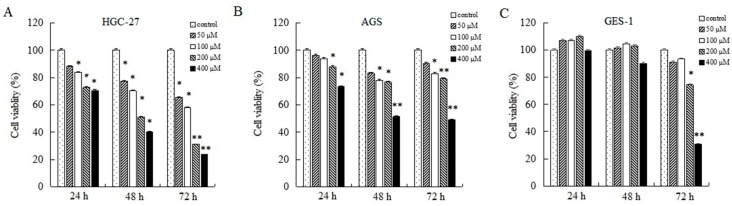
Inhibitory effects of AT-II on cancer cell growth. (**A**) HGC-27, (**B**) AGS and (**C**) GES-1 cells were treated with AT-II at different concentrations for 24 h, 48 h and 72 h. Cell viability was examined by CCK-8 assay. All data were obtained from three independent experiments and expressed as mean ± SD. * *p* < 0.05 and ** *p* < 0.01 vs. control group.

To research the effects of AT-II on cell growth, CCK-8 assays were used to determine relative cell viability. As shown in Figure 1, AT-II treatment groups showed significant inhibitory effects on HGC-27 and AGS cells compared to control group in a concentration and time-dependent manner. Moreover, HGC-27 cells are more sensitive than AGS cells to AT-II. When HGC-27 cells were exposed to 200 μM of AT-II for 48 h, the cell viability reduced to nearly 50%, while AGS needed 400 μM of AT-II treatment. However, even if treated with 400 μM of AT-II for 48 h, it had no cytotoxicity on human normal gastric mucosal epithelium GES-1 cells.

### 2.2. AT-II Affects Morphological Changes

**Figure 2:**
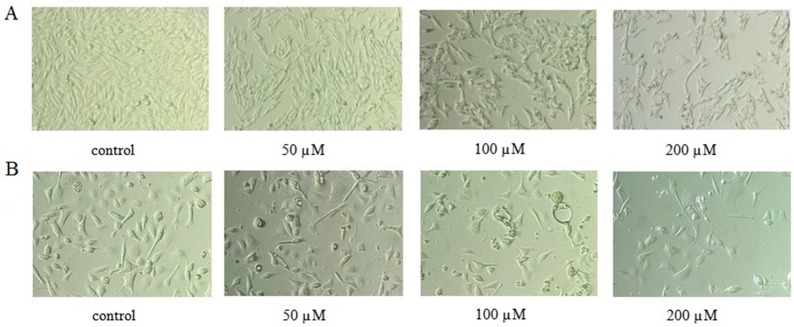
Morphological changes of HGC-27 and AGS cells treated with AT-II for 48 h and observed with an inverted microscope ×200 magnification. (**A**) HGC-27 cells; (**B**) AGS cells.

After being treated with AT-II for 48 h, the morphological changes of HGC-27 and AGS cells were observed with an inverted microscope, which had remarkable differences from the control group. Compared to control group, the majority of HGC-27 and AGS cells treated with AT-II were obviously reduced, distorted and grew slowly. In addition, with a high dose of AT-II treatment, the cell membrane became rough and emerged blebbing and swelling (Figure 2).

### 2.3. AT-II Induces Apoptosis in HGC-27 and AGS Cells

**Figure 3:**
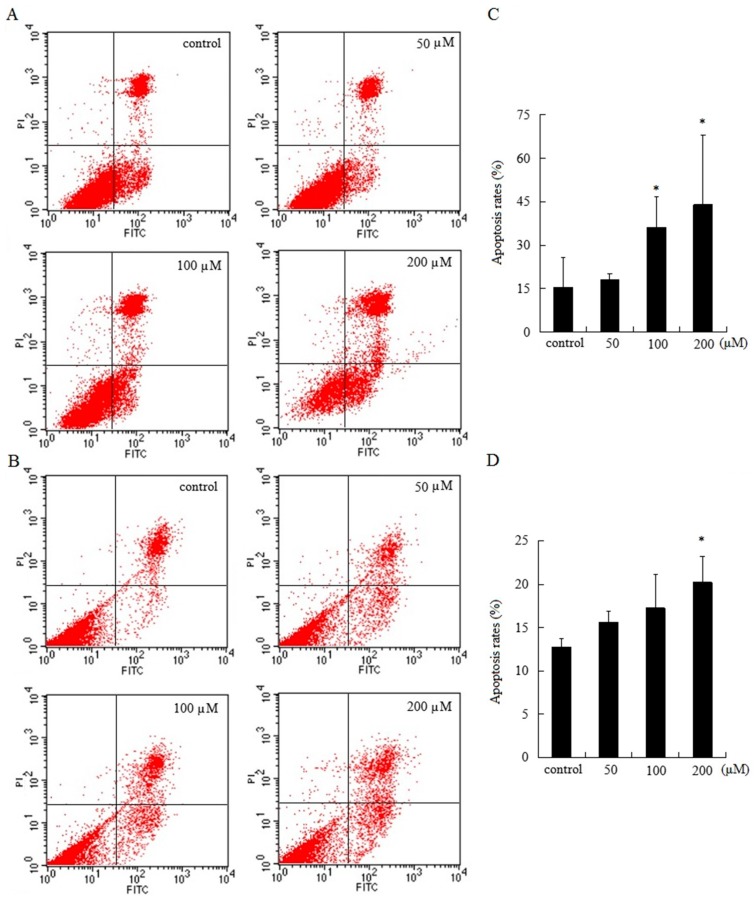
Apoptotic effects of AT-II on HGC-27 and AGS cells for 48 h. (**A**) HGC-27 cells; (**B**) AGS cells; (**C**) the percentages of apoptotic cells in HGC-27 cells; (**D**) the percentages of apoptotic cells in AGS cells. All experiments were performed in triplicates and expressed as mean ± SD. * *p* < 0.05 vs. control group.

HGC-27 and AGS cells were treated with various doses of AT-II for 48 h and stained with Annexin V-FITC/Propidium Iodide (PI). Flow cytometry results demonstrated that cell apoptosis rates of HGC-27 and AGS cells were positively correlated with the concentration of AT-II. The upper right quadrant represented late apoptotic cells and the lower right quadrant represented early apoptotic cells. Treated with 50 μM of AT-II, cell apoptosis rate did not have differences from control group in HGC-27 cells, but the percentages of apoptotic cells were significantly increased with the increasing AT-II concentrations (Figure 3A). However, AGS cells were less sensitive to AT-II than HGC-27 cells and when only exposed to 200 μM of AT-II, AGS cells could exhibit remarkable apoptosis (Figure 3B).

### 2.4. AT-II Suppresses the Capability of Cell Motility

**Figure 4:**
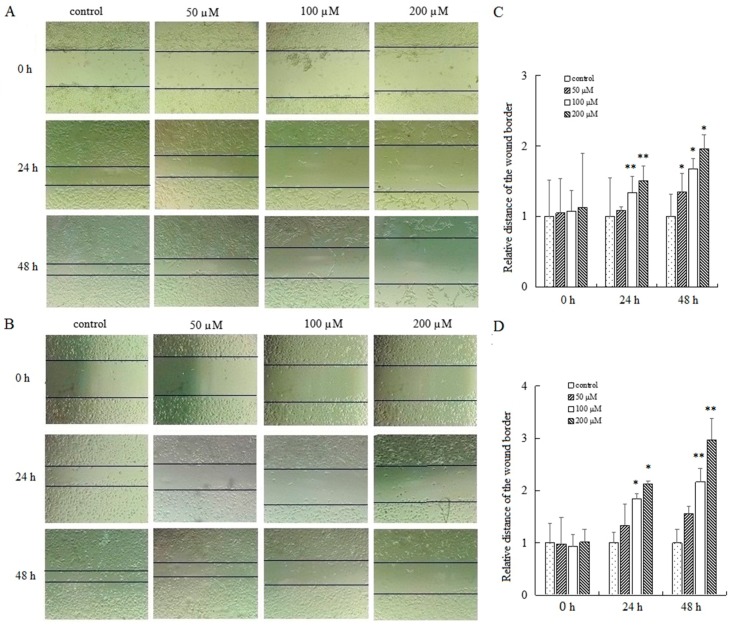
Examination of the motility of HGC-27 and AGS cells by wound healing assay. (**A**) HGC-27 cells; (**B**) AGS cells; (**C**) the relative distances of the wound border in HGC-27 cells; (**D**) the relative distances of the wound border in AGS cells. All experiments were performed three times and expressed as mean ± SD. * *p* < 0.05 and ** *p* < 0.01 vs. control group.

To determine the effect of AT-II on cell migration, cells were exposed to AT-II with different concentrations for 0 h, 24 h and 48 h and wound healing assays were applied to detect the relative migration distance. The results showed a striking difference in cell mobility between control group and AT-II treatment groups. Cells in AT-II treatment groups migrated more slowly. In addition, as time and dose increased, the differences of migration rate were gradually significant between the four groups (Figure 4).

### 2.5. AT-II Regulates the Protein Expression Levels of Bax and Bcl-2

**Figure 5:**
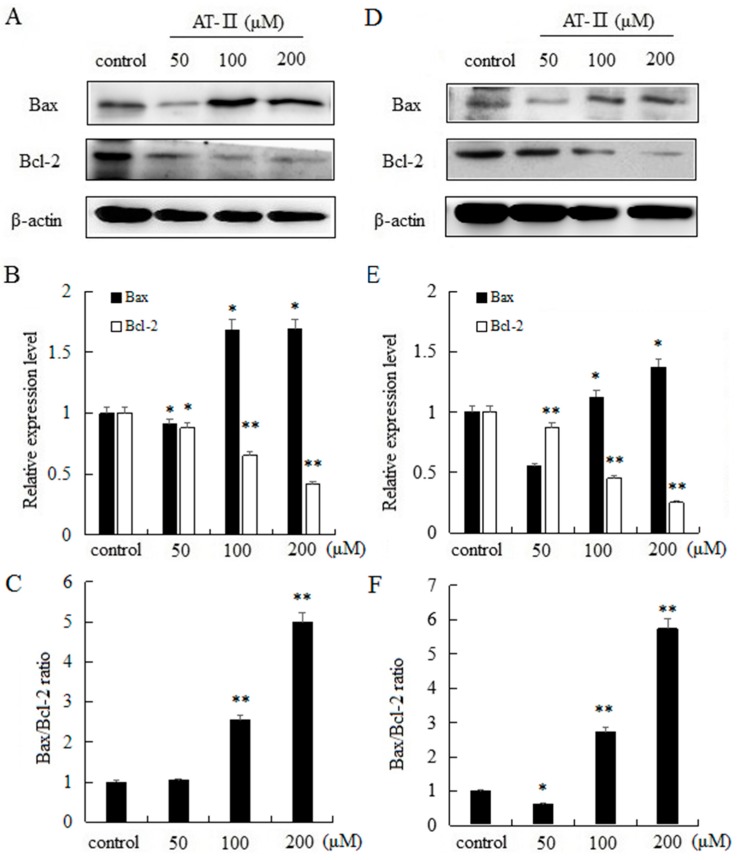
Expression levels of Bax and Bcl-2 and Bax/Bcl-2 ratio in HGC-27 and AGS cells. (**A**) HGC-27 cells; (**B**) relative expression levels of Bax and Bcl-2 in HGC-27 cells; (**C**) Bax/Bcl-2 ratio in HGC-27 cells; (**D**) AGS cells; (**E**) relative expression levels of Bax and Bcl-2 in AGS cells; (**F**) Bax/Bcl-2 ratio in AGS cells. * *p* < 0.05 and ** *p* < 0.01 vs. control group.

The intrinsic apoptosis pathway is mostly regulated by Bcl-2 family proteins and the pro-apoptotic protein Bax and the anti-apoptotic protein Bcl-2 are typically involved in apoptosis and cellular proliferation [19]. Moreover, the ratio of Bax and Bcl-2 has a key role in the development of apoptosis. Our results indicated that AT-II can significantly upregulate the expression level of Bax, and significantly downregulate the expression level of Bcl-2 in a dose-dependent manner in two cell lines. In addition, as the dose of AT-II increased, the ratio of Bax/Bcl-2 also increased (Figure 5).

### 2.6. AT-II Inhibits the Phosphorylation of ERK and Akt in HGC-27 and AGS Cells

**Figure 6:**
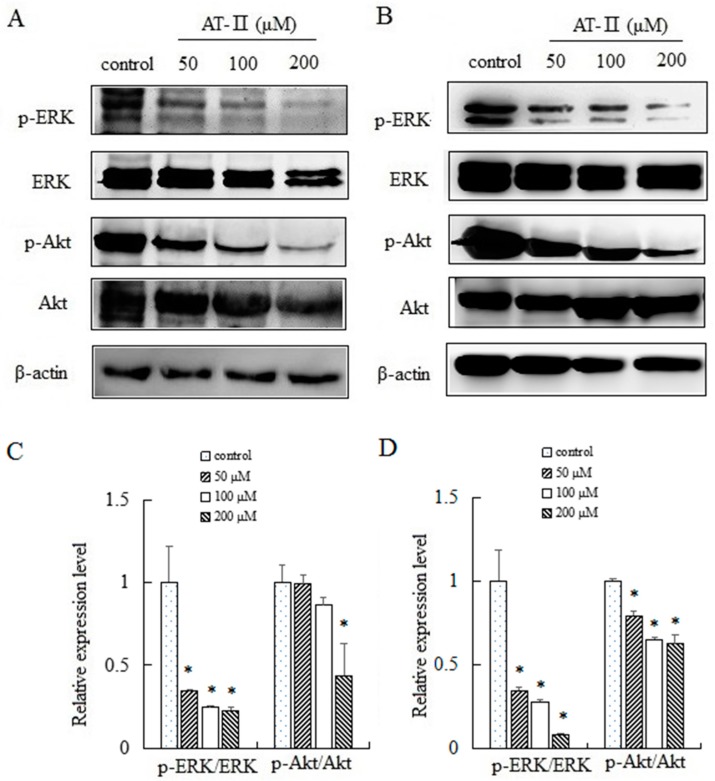
Expression levels of ERK, p-ERK, Akt and p-Akt in HGC-27 and AGS cells. (**A**) HGC-27 cells; (**B**) AGS cells; (**C**) relative expression levels of p-ERK/ERK and p-Akt/Akt in HGC-27 cells; (**D**) relative expression levels of p-ERK/ERK and p-Akt/Akt in AGS cells. * *p* < 0.05 vs. control group.

ERK and Akt played an important role in cell proliferation, apoptosis, migration and staying cell morphology. ERK and Akt were activated through phosphorylating tyr202/204 and ser473 residues, respectively, and exerted their functions [20,21]. We evaluated the expression levels of total ERK and p-ERK by Western blot after treated with AT-II for 48 h. The activation level of ERK was downregulated significantly by AT-II in a dose-dependent manner. Furthermore, we also analyzed the activation pattern of another signaling protein Akt. As shown in Figure 6, a downregulation in the activation of p-Akt was observed after AT-II treatment.

## 3. Discussion

Cell apoptosis, which was involved in physiological growth regulation and tissue homeostasis is a complex and multistage process containing many genes [22,23]. So far, inducing apoptosis is considered as one of the best strategies in cancer therapy. In this study, the effects of AT-II on cell apoptosis were detected by Annexin V-FITC and PI staining. Annexin V-FITC could especially bind to phosphatidylserine, which can translocate from the inner side of the cell membrane to the outer side of the cell membrane during early cell apoptosis. Thus, Annexin V-FITC was used to evaluate the early apoptotic cells and PI was used to evaluate late apoptotic cells and necrotic cells. Flow cytometry results showed that the percentage of apoptotic cells were significantly increased and positively related to the concentrations of AT-II. However, the effects of AT-II on apoptosis in HGC-27 and AGS cells are different. HGC-27 cells are more sensitive than AGS cells to AT-II. AT-II could induce apoptosis in HGC-27 cells when exposed to 100 μM, while AGS cells were induced apoptosis only when the concentration of AT-II reached to 200 μM. The differences between HGC-27 cells and AGS cells may be because of differentiated stage. HGC-27 cells are undifferentiated gastric carcinoma cell, while AGS cells are derived from untreated tumor fragments belong to poor differentiated gastric carcinoma cells. At the same time, mitochondria-mediated apoptosis pathway is well understood and Bcl-2 family proteins play an important role in controlling mitochondrial pathway. Bax is pro-apoptotic protein, which could interact with voltage-dependent ion channel of mitochondria membrane and induce the release of cytochrome C. In contrast, Bcl-2 is anti-apoptotic protein, which prevents the release of cytochrome C from mitochondria to cytoplasm [24,25,26]. Consistent with the above results, Western blot analysis revealed AT-II treatment resulted in the upregulation of Bax, the downregulation of Bcl-2 and an increase of the Bax/Bcl-2 ratio in both two cell lines. These data suggested that AT-II induced mitochondrial dependent apoptosis pathway though regulating apoptosis related proteins and the ratio of Bax/Bcl-2.

Cancer metastasis is the major cause of morbidity and mortality in millions of patients with cancer, and the migration of cancer cells is one of the important steps during the invasion. If an agent can inhibit proliferation and migration of tumor cells, it might be hopeful to suppress cancer progression and metastasis and reduce death rates [27]. In our research, we found that AT-II was able to inhibit cell proliferation and motility in a concentration and time-dependent manner in HGC-27 and AGS cells, indicating that AT-II may inhibit metastasis of invasive gastric cells. 

Ras/ERK and PI3K/Akt signaling pathways are vital for cell proliferation, differentiation, survival, motility, metabolism, tumor development and drug resistant. It is reported that both two signaling pathways are constantly activated in many cancers [28,29]. ERK is phosphorylated and activated by mitogen though the Ras/Raf/MEK signaling pathway. Then, p-ERK stimulates the transcription of some related proteins and is involved in cell proliferation, apoptosis and migration [30,31]. Akt, as an important downstream effecter of PI3K, is activated by phosphorylating. Activated Akt phosphorylats several downstream targets of the survival and apoptotic pathways and exerts its functions [32,33]. Our results demonstrated that AT-II treatment resulted in decreasing the levels of p-ERK and p-Akt, which was accompanied by the downregulation of Bcl-2. The data suggested that AT-II may, through modulating ERK and Akt signaling pathways, inhibit cell proliferation, motility and induce apoptosis.

In summary, our results indicate that AT-II exerts anti-cancer effects by inhibiting proliferation, motility and inducing apoptosis and may be due to inactivating Ras/ERK and PI3K/Akt signaling pathways, which might shed light on therapy of gastric carcinoma.

## 4. Materials and Methods

### 4.1. Reagents

AT-II (purity ≥ 98%) was purchased from Must Bio-technology Co., Ltd. (Chengdu, China). Dimethyl sulfoxide (DMSO) was purchased from Sigma (St. Louis, MO, USA). CCK-8 Kit and human Bcl-2, Bax, Akt, p-Akt, ERK, p-ERK and β-actin polyclonal antibodies were purchased from Beyotime (Shanghai, China). Annexin V-FITC Apoptosis Detection Kit was purchased from KeyGEN BioTECH (Nanjing, China).

### 4.2. Cell Lines and Cell Culture

Human gastric cancer cell lines HGC-27 and AGS were purchased from Cell Bank of Type Culture Collection of Chinese Academy of Sciences (Shanghai, China). Human normal gastric mucosal epithelium cell lines GES-1 was supplied by The First Hospital of China Medical University (Shenyang, China). All cells were cultured in RPMI-1640 medium supplemented with 10% fetal bovine serum (FBS), 100 U/mL penicillin and 100 μg/mL streptomycin at 37 °C. 

### 4.3. Cell Growth Assay

CCK-8 assay was used to determine relative cell viability after AT-II treatment for 24 h, 48 h and 72 h, respectively. HGC-27, AGS and GES-1 cells were seeded in 96-well plates at a density of 3 × 10^3^ cells/well and treated with AT-II diluted by complete medium at serial concentrations (0/50/100/200/400 μM) on the next day. CCK-8 solution of 10 μL was added to each well and cells were incubated for another 1 h at 37 °C. Then, the optical density was measured at 450 nm (A_450_). 

### 4.4. Cell Morphological Assessment

HGC-27 and AGS cells were seeded in 24-well plates and treated with different concentrations of AT-II (0/50/100/200 μM) for 48 h. Then, the morphological changes of cells were observed under an inverted microscope (Olympus, Tokyo, Japan).

### 4.5. Apoptosis Assay

Cell apoptosis rates were detected by flow cytometry tests. HGC-27 and AGS cells were seeded in 6-well plates (2 × 10^5^ cells/well) and, on the following day, were exposed to AT-II with 0, 50, 100 and 200 μM for 48 h. Cells were collected, rinsed with PBS, re-suspended in binding buffer and incubated with Annexin V-FITC and PI for 5–15 min at room temperature in the dark and then samples were analyzed within 1 h by FACS Calibur (BD Biosciences, Shanghai, China).

### 4.6. Wound Healing Assay

The motility and spreading capabilities of the cells were calculated by wound healing assay. HGC-27 and AGS cells were seeded in 24-well plates (5 × 10^4^ cells/well) and cultured to near (>90%) confluence. A sterile 200 μL pipette tip was used to scratch a separate wound through cell monolayers. Then, the medium was removed and replaced with fresh medium containing 0, 50, 100 and 200 μM of AT-II. Pictures were taken to ensure that the line just appears in each picture at 0 h, 24 h and 48 h, respectively.

### 4.7. Western Blot Analysis

HGC-27 and AGS cells were seeded in 6-well plates (2 × 10^5^ cells/well) and were treated with different doses of AT-II on the following day. After 48 h, total protein was extracted by Radio Immunoprecipitation Assay (RIPA) buffer (1 mM MgCl_2_, 10 mM Tris-HCl, pH 7.4, 1% Triton X-100, 0.1% SDS and 1% NP-40). Protein expression level was analyzed by Western blot. Proteins (25 μg/well) were separated on 10% SDS-PAGE gels and transferred to PVDF membranes. After that, the membranes were blocked in 1% bovine serum albumin (BSA) for 1 h at room temperature and incubated overnight with primary antibodies of target proteins at 4 °C. With β-actin as a loading control, the expression levels of Bax, Bcl-2, Akt, p-Akt, ERK and p-ERK were evaluated out and quantified by Labworks 4.0 software.

### 4.8. Statistical Analysis

All experiments were performed three times and the results were represented as mean ± standard deviation (SD). Statistical analysis used the Duncan test and one-way analysis of variance (ANOVA), with *p* < 0.05 considered to be significant and *p* < 0.01 considered to be significant remarkably.
